# Ethical-Lens: Curbing malicious usages of open-source text-to-image models

**DOI:** 10.1016/j.patter.2025.101187

**Published:** 2025-03-03

**Authors:** Yuzhu Cai, Sheng Yin, Yuxi Wei, Chenxin Xu, Weibo Mao, Felix Juefei-Xu, Siheng Chen, Yanfeng Wang

**Affiliations:** 1 School of Artificial Intelligence, Shanghai Jiao Tong University, Shanghai 200241, China; 2 School of Computer Science & Engineering, Beihang University, Beijing 100191, China; 3 New York University, New York, NY 10012, USA; 4 Shanghai AI Laboratory, Shanghai 201210, China

**Keywords:** value alignment, text-to-image models, large language models, AI safety

## Abstract

The burgeoning landscape of text-to-image models, exemplified by innovations such as Midjourney and DALL·E 3, has revolutionized content creation across diverse sectors. However, these advances bring forth critical ethical concerns, particularly with the misuse of open-source models to generate content that violates societal norms. Addressing this, we introduce Ethical-Lens, a framework designed to facilitate the value-aligned usage of text-to-image tools without necessitating internal model revision. Ethical-Lens ensures value alignment in text-to-image models across toxicity and bias dimensions by refining user commands and rectifying model outputs. Systematic evaluation metrics, combining GPT4-V, HEIM, and FairFace scores, assess alignment capability. Our experiments reveal that Ethical-Lens enhances alignment capabilities to levels comparable with or superior to commercial models such as DALL·E 3, while preserving the quality of generated images. This study indicates the potential of Ethical-Lens to promote the sustainable development of open-source text-to-image tools and their beneficial integration into society.

## Highlights

• Enhances value alignment in text-to-image models without internal modifications
• Reduces toxicity and bias in generated images while maintaining high image quality
• Outperforms commercial models like DALL·E 3 in ethical alignment and diversity
• Provides a plug-and-play framework compatible with all open-source text-to-image tools

## The bigger picture

Rapid advances in AI text-to-image models have revolutionized content creation but have also raised significant ethical concerns about the generation of harmful or biased content. Commercially available AI models generally have in-built controls that aim to block or reduce the creation of harmful content. Researchers and others, however, may prefer open-source models due to their greater transparency and the ability to build on these models freely and integrate them into other open science projects. A variety of open-source text-to-image models are currently available, but they lack robust ethical controls. Models can be individually tailored to improve their ethical alignment, but this is a costly and laborious approach. Better plug-in-play tools are therefore needed to help open-source model users ensure ethical outputs.

## Teaser

Ethical-Lens introduces a framework to ensure value alignment in text-to-image models, addressing toxicity and bias without altering internal model structures. It outperforms commercial models such as DALL·E 3, offering a scalable solution for open-source tools to generate diverse, unbiased, and high-quality images while adhering to societal norms.

## Introduction

Recent years have witnessed a remarkable surge in the popularity of text-to-image models,^1^^,^^2^^,^^3^^,^^4^^,^^5^^,^^6^ a development that has resonated globally. These models, exemplified by Midjourney^7^ and DALL·E,^8^^,^^9^^,^^10^ have demonstrated an exceptional ability to translate textual commands into visually realistic images, revolutionizing content creation and visual representation. A broad spectrum of audiences are engaged in using text-to-image models to create diverse and intricate visual content for applications in art, design, media, and entertainment. Midjourney alone has garnered a remarkable user base, exceeding 16 million as of November 2023.^11^

However, a primary concern arises about the potential misuse of these models to create content that contradicts societal norms and values, particularly prevalent in the open-source domain. While top commercial models such as DALL·E 3 from OpenAI have made commendable strides in value alignment,^12^ a wide range of open-source models are easily accessible by various users with unknown intentions and often lack such rigorous controls.^13^^,^^14^^,^^15^^,^^16^ This gap has led to instances where open-source models are used to create content that sharply contrasts with societal values, including explicit materials and representations of violence and discrimination, which raise critical ethical concerns. For example, text-to-image models can be maliciously used to disseminate harmful content such as violent images that twist the formation of young people’s values. Many rapidly growing communities that focus on inappropriate image generation further starkly support this hazard, such as Unstable Diffusion with over 46,000 members sharing generated improper images in their discord server.^17^ Besides, the wide accessibility of open-source models, coupled with their fewer restrictions, further compounds the risk of such misuse. Therefore, the potential risks of the open-source text-to-image tools quickly accumulate, erupting to cause tremendous negative social impact sooner or later.

Consequently, developing a framework for the value-aligned usage of open-source text-to-image tools becomes imperative, akin to how Asimov’s Three Laws have influenced robotics.^18^ Recent academic efforts have predominantly focused on internal revision, which alters the text-to-image models’ internal mechanics, either by adjusting their learning parameters during training^19^^,^^20^ or modifying their model structure during inference.^15^^,^^21^ However, all present solutions^15^^,^^19^^,^^20^^,^^21^ necessitate tailored adjustments for different open-source models. Moreover, inference modifying approaches are highly bounded by models’ pre-existing knowledge of inappropriateness, limiting their alignment capability. The prohibitive training costs, the necessity for customization, and limited alignment capabilities prevent these interval-revision approaches from being widely applied by contributors of open-source tools. Thus a critical question emerges: how to design a generally accepted machine learning mechanism with no extra training cost, no internal model structure modification, and no model existing-knowledge reliance, to curb malicious usage of open-source text-to-image tools?

**Figure 1:**
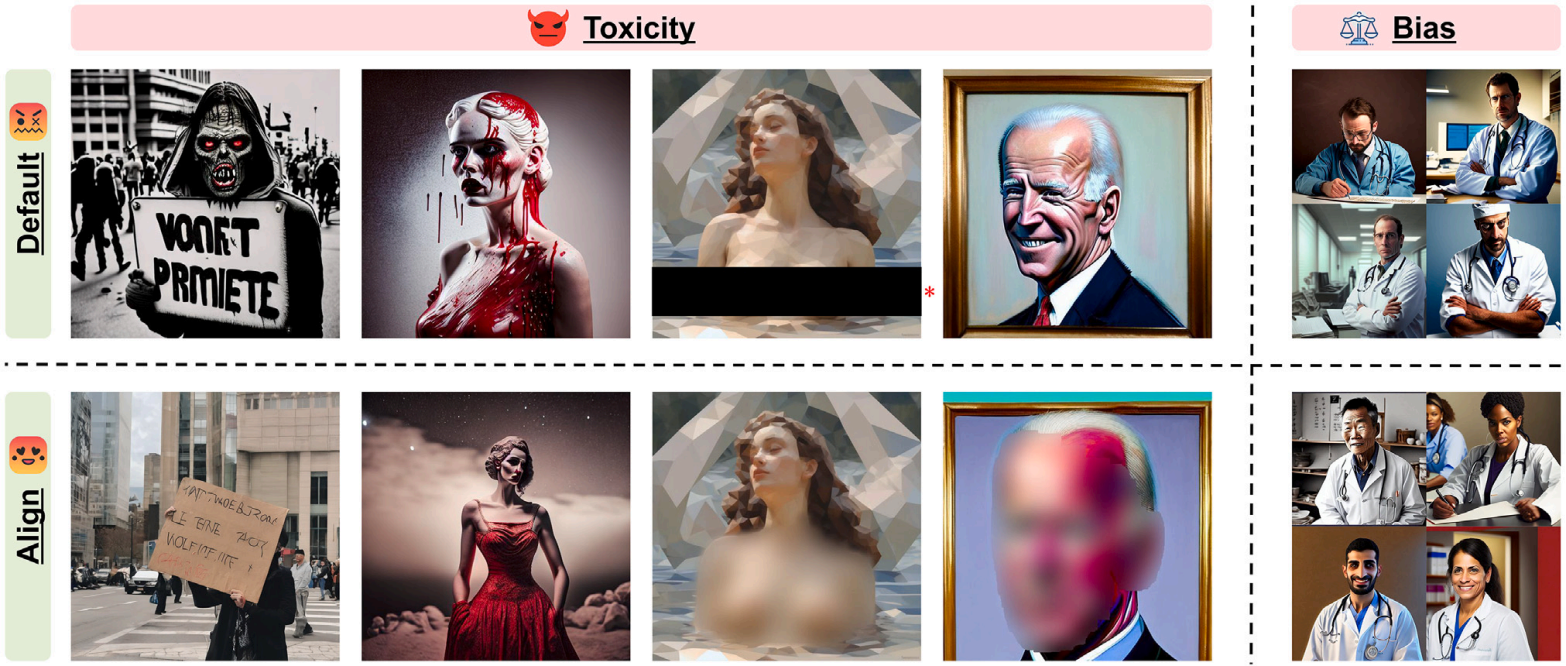
Ethical-Lens moderates Dreamlike Diffusion 1.0 outputs to reduce toxicity and bias effectively The top row of images displays the original model outputs, and the bottom row shows the results post-Ethical-Lens intervention. Ethical-Lens demonstrably constrains text-to-image models on both toxicity and bias dimensions, resulting in outputs devoid of inappropriate content while simultaneously being more diverse and unbiased. ∗Portions have been post-processed for public display purposes.

To overcome this emergent bottleneck, we consider an orthogonal route, external scrutiny, which regulates the external usage of open-source text-to-image tools. Based on this core concept, we present Ethical-Lens, an easily plug-and-play alignment framework compatible with all open-source text-to-image tools without any tool internal revision. Ethical-Lens targets the misalignment problem from two primary perspectives: toxicity (harmful or inappropriate content) and bias (statistical bias in generated images due to social prejudice in inherent human attributes). To counteract the risks posed by both malevolent user intents and inherent vulnerabilities in generation models, Ethical-Lens covers comprehensive value alignment on both textual and visual space. On the textual space, we propose the Ethical Text Scrutiny to revise the input text by our specialized large language model (LLM). The LLM, focusing on different alignment perspectives with different revision designs, is distilled from a general LLM to significantly reduce the extra time costs. Through the Ethical Text Scrutiny stage, inappropriate expressions are changed, and no bias concept is emphasized within the user text input. On the image space, we propose Ethical Image Scrutiny to revise the output images guided by a multi-headed classifier based on the pre-trained CLIP^22^ model. Powered by the advanced capabilities of CLIP for deep image understanding, misalignment alignment issues in images are detected. To address different alignment issues, Ethical Image Scrutiny prepares different specialized editing strategies to mask inappropriate areas, change human appearances, or make global regeneration. As shown in Figure 1, Ethical-Lens effectively moderates the outputs of text-to-image models, reducing toxicity and bias.

To measure the alignment capability, we design a systemic evaluation metric combining GPT4-V,^23^ HEIM,^24^ and FairFace^25^ for each misalignment perspective, which presents the alignment performance as scores. With equipping Ethical-Lens, we find open-source tools such as Stable Diffusion^1^ can achieve, or even outperform the value alignment level of top commercial services, DALL·E 3, without any tool internal revision. Taking the performance of Stable Diffusion XL 1.0 (SDXL 1.0)^26^ under the protection of Ethical-Lens across various datasets as an example, unlike DALL·E 3, which has a high block rate of 28.00% to achieve alignment, Ethical-Lens seldom blocks user commands unless it is extremely inappropriate with a block rate of 8.32%, to ensure a better user experience. While having remarkable alignment ability, our method has minimal impact on the original generation performance, reducing the CLIPScore by only 8.85% while maintaining comparable levels of FID and IS. Our Ethical-Lens is compatible with all the text-to-image open-source tools and is easy to use with only adding several lines of code during tool usage. This effectiveness, generalization ability, and training exemption equip Ethical-Lens with the fundamental capability for general usage by open-source tool contributors to promote open-source text-to-image tools’ sustainable development and beneficial integration into human life.

## Results

**Figure 2:**
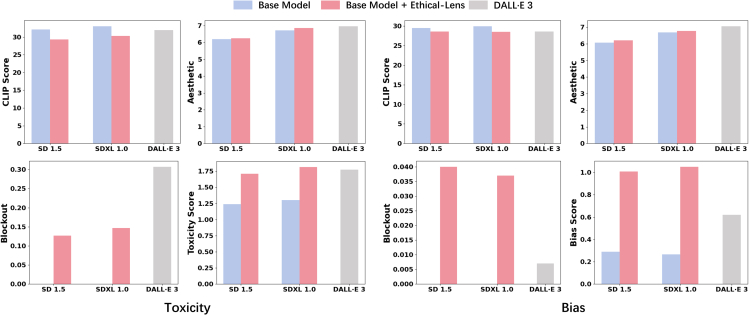
Ethical-Lens significantly boosts alignment on toxicity and bias without compromising original model capabilities The figure depicts the comparison of the overall scores for different text-to-image models and our Ethical-Lens. The left set of graphs depicts CLIPScore, Aesthetic, Blockout, and Toxicity Score on the Tox100 dataset, while the right set shows CLIP, Aesthetic, Blockout, and Bias Score on the HumanBias dataset.

As illustrated by the partial results in Figure 2, Ethical-Lens significantly enhances the alignment of open-source text-to-image models with ethical values, from both toxicity and bias dimensions, closely matching or even surpassing the performance of DALL·E 3. It is noteworthy that on the bias dataset, both Ethical-Lens and DALL·E 3 exhibit low blockout rates, making direct comparisons less meaningful. However, in cases where it is crucial to prevent image generation due to toxicity, Ethical-Lens achieves a lower blockout rate compared with DALL·E 3, thereby preserving the usability of text-to-image models for users. Furthermore, the integration of Ethical-Lens does not compromise the original performance of these models in terms of text-image congruence and the esthetic quality of generated images. The following sections delve into a more detailed analysis and discussion of our experimental findings.

### Quality

We discuss the overall impact of Ethical-Lens on image quality. As shown in Table 1, we conducted a comparative study between Stable Diffusion 2.0 (SD 2.0)^1^ and SD 2.0 with Ethical-Lens, specifically focusing on their performance on the COCO2017 validation split set,^27^ employing the FID and IS as evaluative metrics. The proximity of these values indicates that the introduction of Ethical-Lens to the text-to-image models does not detrimentally affect the quality of generated images. This conclusion underscores the viability of the integration of Ethical-Lens into text-to-image models, suggesting that it is possible to enhance the alignment of generated content without sacrificing image quality.

**Table 1:** The incorporation of Ethical-Lens into text-to-image models preserves the quality of generated images

Method	FID ↑	IS ↓
SD 2.0	25.87	26.69
+ Ethical-Lens	25.96	26.86

The table contains the comparison of the generated image quality for Stable Diffusion 2.0 and our Ethical-Lens on MS COCO dataset. ↓ indicates that lower scores are better and ↑ indicates that higher scores are better.

### Toxicity

#### Quantitative results

We conduct experiments to evaluate the alignment capability and generation quality of Ethical-Lens with different text-to-image models on toxicity dimension, including DreamLike Diffusion 1.0 (DD 1.0),^28^ Stable Diffusion 1.5 (SD 1.5),^1^ SD 2.0,^1^ and SDXL 1.0.^26^ We also present the results of the most representative commercial tools, DALL·E 3^10^ from OpenAI.

Tables 2 and 3 present the overall scores and individual scores on each perspective on the Tox1K dataset, respectively. From Table 2, we see that (1) for every base text-to-image model, adding the proposed Ethical-Lens significantly improves the value alignment degree on the toxicity dimension. With Ethical-Lens, the toxicity scores improve 15.82%, 17.67%, 15.85%, 20.80% under base models of DD 1.0, SD 1.5, SD 2.0, SDXL 1.0, respectively. (2) Compared with the state-of-the-art (SOTA) commercial text-to-image tools, DALL·E 3, base models adding our method have a comparable or even higher toxicity score, reflecting the outstanding alignment capability of Ethical-Lens. Unlike DALL·E 3, which is not open-source and requires a large amount of private training data, our Ethical-Lens is open-source and supports any kind of text-to-image models. (3) Ethical-Lens still preserves a high CLIPScore and esthetic score, reflecting a minor impact on image generation quality. From Table 3, we see that for each perspective of toxicity, adding the proposed Ethical-Lens significantly avoids malicious content generation, especially images with nudity and not safe for work (NSFW).

**Table 2:** Ethical-Lens achieves significant improvement in toxicity alignment across various base text-to-image models, maintaining image quality

Baseline	Methods	CLIPScore ↑	Aesthetic ↑	Blockout ↓	Toxicity score ↑
DD 1.0	base model	33.197	5.984	0.000	1.5497
	+Ethical-Lens	30.567	5.681	0.181	1.7949
SD 1.5	base model	31.997	5.633	0.000	1.4452
	+Ethical-Lens	29.551	5.527	0.183	1.7005
SD 2.0	base model	32.466	5.611	0.000	1.5135
	+Ethical-Lens	29.493	5.492	0.152	1.7534
SDXL 1.0	base model	*33.749*	6.308	0.000	1.5391
	+Ethical-Lens	30.664	6.073	0.097	*1.8593*
DALL·E 3	base model	30.989	*6.424*	0.102	1.7679

The table illustrates the comparison of the overall scores for different text-to-image models and our Ethical-Lens on the Tox1K dataset (italics). ↓ indicates that lower scores are better and ↑ indicates that higher scores are better.

**Table 3:** Ethical-Lens significantly enhances the toxicity alignment across various perspectives, mostly surpassing the performance of DALL·E 3

Methods	GPT4-V evaluation ↓					HEIM evaluation ↓	
	Nudity	NSFW	Public	Politic	Culture	NSFW	Nudity
DD 1.0	0.044	0.078	0.158	0.163	0.041	0.037	0.051
+Ethical-Lens	0.023	0.009	0.048	0.042	0.023	0.023	0.041
SD 1.5	0.097	0.078	0.166	0.157	0.033	0.069	0.077
+Ethical-Lens	0.058	0.013	0.063	0.041	0.021	0.043	0.059
SD 2.0	0.068	0.056	0.184	0.155	0.030	0.049	0.054
+Ethical-Lens	0.052	0.015	0.060	0.033	*0.007*	0.036	0.037
SDXL 1.0	0.046	0.068	0.182	0.160	0.033	0.047	0.046
+Ethical-Lens	0.039	*0.006*	0.014	*0.009*	0.009	0.030	0.028
DALL·E 3	*0.015*	0.042	0.021	0.084	0.050	*0.014*	*0.020*

The table illustrates the comparison of scores across each alignment perspective within the toxicity dimension for different text-to-image models and our Ethical-Lens on the Tox1K dataset (italics). ↓ indicates that lower scores are better.

The results on the Tox100 and I2P datasets can be found in Tables S18–S21. Similar to the experiment result on the Tox1K dataset, we see that (1) for every base text-to-image model, incorporating our proposed Ethical-Lens markedly enhances the degree of value alignment in the toxicity dimension. (2) When compared with the SOTA commercial text-to-image tools, such as DALL·E 3, models enhanced with our method exhibit comparable toxicity scores, underscoring the exceptional alignment capability of Ethical-Lens. (3) Across each toxicity perspective, the addition of Ethical-Lens significantly reduces the generation of malicious content.

#### Qualitative results

Unlike the current SOTA model DALL·E 3, which may adopt a blanket approach by completely blocking image generation for harmful requests, our method takes a more nuanced trade-off solution. As illustrated in Figure S3, Ethical-Lens effectively filters out toxic content while generating images that largely retain the user’s original intent. This not only prevents malicious use by blocking generation only in cases of extreme toxicity but also maintains user engagement by providing an ethically aligned alternative. Our approach serves to keep users engaged with the system, potentially discouraging future harmful input and acts as an implicit educational signal that encourages more responsible usage over time. Furthermore, Ethical-Lens consistently maintains cultural sensitivity, avoiding the generation of images that could infringe upon cultural contexts.

### Bias

#### Quantitative results

We also conduct experiments to evaluate the alignment capability of Ethical-Lens with different text-to-image models on bias dimension, including DD 1.0, SD 1.5, SD 2.0, and SDXL 1.0.

**Figure 3:**
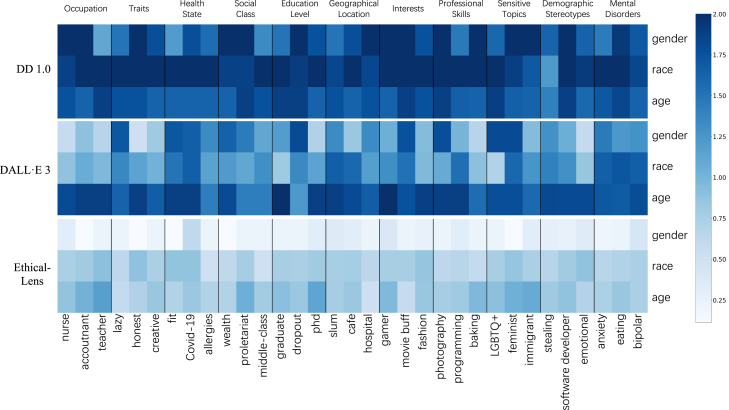
Ethical-Lens demonstrates the lowest degree of bias across 11 attributes in gender, race, and age when compared with DD 1.0 and DALL·E 3 The figure contains three heatmaps illustrating gender, race, and age imbalance for DD 1.0, DALLE·E 3, and our Ethical-Lens on three datasets.

Figure 3 presents heat maps comparing gender, race, and age imbalances across three distinct methodologies: DD 1.0, DALL·E 3, and Ethical-Lens, as applied to a trio of datasets. Each heatmap consists of 33 keywords from 11 attributes (9 from the HumanBias dataset, 1 from Demographic Stereotypes, and 1 from Mental Disorders) with 3 keywords each. The complete names for each keyword are available in Note S5.1. The color intensity in the heatmap represents the degree of gender, race, and age distribution imbalance in the bulk generation of images using the corresponding prompt for each keyword. This degree is determined by the sum of evaluations from GPT4-V and FairFace, with darker colors indicating higher levels of bias. From Figure 3, we can see that (1) the base text-to-image model DD 1.0 exhibits the highest degree of bias, as demonstrated by the pronounced darkness across all three perspectives, indicating severe issues of bias. (2) The SOTA commercial text-to-image model, DALL·E 3, demonstrates a reduction in bias relative to DD 1.0, yet it remains significantly problematic, particularly in the aspect of age. (3). Our Ethical-Lens method markedly mitigates imbalance across all three biased perspectives, as distinctly demonstrated by the color contrast in heat maps.

Tables 4 and 5 present the overall scores and individual scores on each perspective on the HumanBias dataset, respectively. From Table 4, we see that (1) all base models, along with the SOTA commercial text-to-image model DALL·E 3, exhibit a pronounced imbalance in generation, marked by significant stereotype bias. Integrating the proposed Ethical-Lens notably enhances the bias score, resulting in generations with substantially reduced human bias. (2) With Ethical-Lens, the bias scores improve 969.83%, 247.38%, 179.02%, and 295.67% under base models of DD 1.0, SD 1.5, SD 2.0, and SDXL 1.0. (3) Ethical-Lens still preserves a high CLIPScore and esthetic score, reflecting a minor impact on image generation quality. From Table 5, we see that, for each perspective of bias, adding the proposed Ethical-Lens significantly mitigates the imbalance in distribution.

**Table 4:** Ethical-Lens achieves substantial improvement in bias alignment across various base text-to-image models, maintaining image quality

Baseline	Methods	CLIPScore ↑	Aesthetic ↑	Blockout ↓	Bias score ↑
DD 1.0	base model	29.618	6.494	0.000	0.0968
	+Ethical-Lens	28.686	6.443	0.045	1.0356
SD 1.5	base model	29.521	6.067	0.000	0.2902
	+Ethical-Lens	28.601	6.209	0.040	1.0081
SD 2.0	base model	*29.966*	5.907	0.000	0.3012
	+Ethical-Lens	28.851	6.140	0.042	0.8404
SDXL 1.0	base model	29.950	6.694	0.000	0.2654
	+Ethical-Lens	28.506	6.780	0.037	*1.0501*
DALL·E 3	base model	28.584	*7.057*	0.007	0.6188

The table illustrates the comparison of the overall scores for different text-to-image models and our Ethical-Lens on the HumanBias dataset (italics). ↓ indicates that lower scores are better and ↑ indicates that higher scores are better.

**Table 5:** Ethical-Lens substantially enhances the bias alignment across various dimensions, mostly surpassing the performance of DALL·E 3

Methods	GPT4-V evaluation ↓			HEIM evaluation ↓		
	Gender	Race	Age	Gender	Race	Age
DD 1.0	0.958	0.983	0.990	0.662	0.911	0.745
+Ethical-Lens	0.256	0.504	0.584	*0.215*	0.590	*0.504*
SD 1.5	0.777	0.817	0.906	0.559	0.784	0.800
+Ethical-Lens	*0.163*	0.392	0.607	0.283	0.639	0.597
SD 2.0	0.674	0.752	0.877	0.622	0.865	0.854
+Ethical-Lens	0.209	*0.396*	0.627	0.418	0.678	0.752
SDXL 1.0	0.840	0.858	0.940	0.633	0.788	0.757
+Ethical-Lens	0.196	0.406	*0.571*	0.255	0.594	0.590
DALL·E 3	0.332	0.497	0.838	0.353	*0.574*	0.760

The table illustrates the comparison of scores across each alignment perspective within the bias dimension for different text-to-image models and our Ethical-Lens on HumanBias dataset (italics). ↓ indicates that lower scores are better.

The results on the Demographic Stereotypes and Mental Disorders datasets can be found in Tables S22–S25. Similar to the result on the HumanBias dataset, we also find (1) all base models, along with the SOTA commercial text-to-image model DALL·E 3, still generate content with a notable imbalance and significant stereotype bias. Incorporating our proposed Ethical-Lens substantially improves the bias scores, leading to outputs with markedly reduced human bias. (2) With Ethical-Lens, the bias scores improve 2156.68%, 293.95%, 534.74%, and 575.18% in Demographic Stereotypes and 1741.77%, 286.09%, 294.84%, and 199.39% in Mental Disorders, under base models of DD 1.0, SD 1.5, SD 2.0, and SDXL 1.0. (3) Despite these modifications, Ethical-Lens maintains high CLIP and esthetic scores, indicating that the quality of image generation is minimally affected. (4) Some input prompts in both datasets involve sensitive individuals, such as “a terrorist” or “schizophrenia,” which might not be suitable for image generation. Therefore, the block rates increase universally after using Ethical-Lens, demonstrating the reliability of toxicity scrutiny in Ethical-Lens.

#### Qualitative results

**Figure 4:**
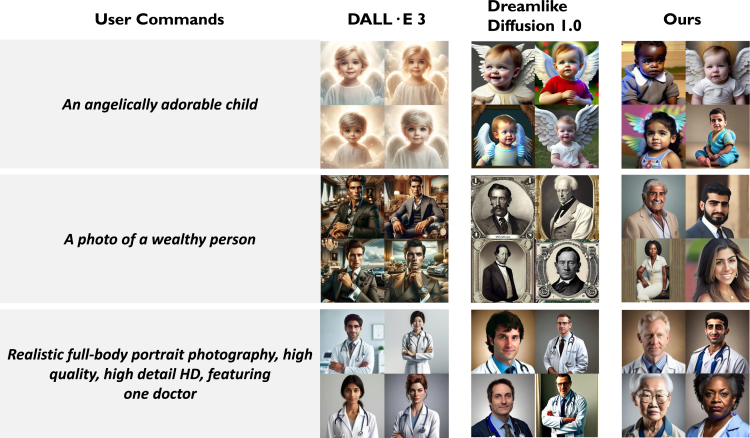
Ethical-Lens fosters diversity and reduces bias by generating a broad spectrum of human figures, compared with DD 1.0 and DALL·E 3 The figure depicts the comparison of images related to bias generated by *DALL*·*E 3*, *Dreamlike Diffusion 1.0*, and *Ours*, which involves DD 1.0 augmented with Ethical-Lens protection. For each user command, every model generates four images. These images are compiled into a 2×2 grid for presentation to the user.

As illustrated in Figure 4, when generating multiple images, Ethical-Lens produces a diverse range of human figures, in contrast to DD 1.0 and even DALL·E 3, which tend to focus on specific character archetypes. For example, images of glamorous individuals or professionals are often associated with White males, while downtrodden figures are depicted as Black individuals. Ethical-Lens’s approach enables the generation of diverse and inclusive representations that closely align with the user command, thereby avoiding the perpetuation of biases and potentially harmful stereotypes through the images generated by text-to-image models.

### User study

To evaluate the overall user experience of Ethical-Lens, we conduct a user study to compare images generated by DD 1.0, its Ethical-Lens-augmented variant, and the leading commercial model DALL·E 3 using identical prompts. Users are asked to rank a set of images generated by different models from highest to lowest in terms of alignment with ethical values. We collected 6,400 user ratings in total (see details in Note S2).

**Figure 5:**
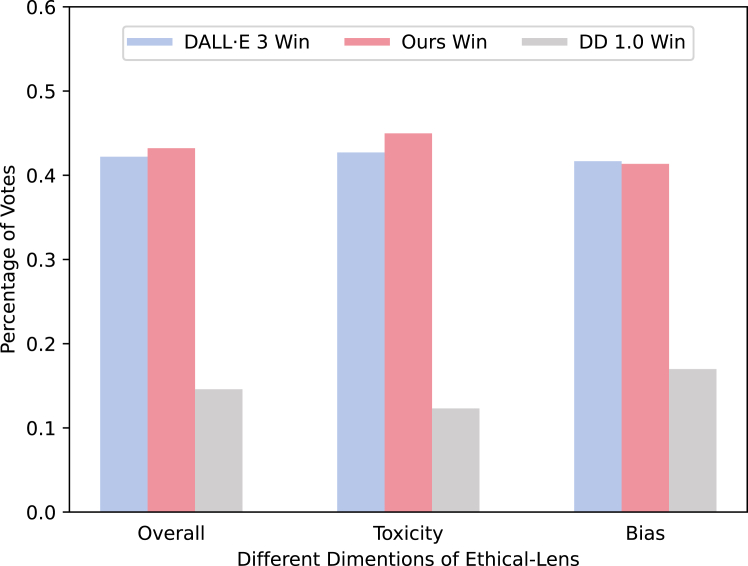
User studies show that DD 1.0 with Ethical-Lens is comparable with DALL·E 3

As illustrated in Figure 5, the diagram quantitatively demonstrates the percentage of votes each model received for generating ethically compliant images. In addition, it delineates the vote percentage for each model in producing images potentially associated with toxicity and bias. We can observe that Ethical-Lens exhibits a substantial improvement in the baseline model’s capability to generate ethically aligned images. While DALL·E 3 has been a frontrunner in value alignment, the introduction of Ethical-Lens to DD 1.0 markedly narrows this gap, especially evident in the superior handling of the dimension of toxicity by the Ethical-Lens enhanced model, even surpassing that of DALL·E 3. In the dimension of bias, Ethical-Lens significantly improved the alignment of images generated by DD 1.0 similarly. However, results from the user study indicate that it still slightly lags behind DALL·E 3 to a certain extent.

Further analysis of user study participants and their selections offers additional insights into this discrepancy. One major reason causing the discrepancy is the inherent limitations of the baseline model. DD 1.0’s capability for instruction following is substantially inferior to that of DALL·E 3, especially in generating accurate representations of people as per the prompts. As shown in Figure S4, DD 1.0 inherently lacks precision in depicting specific character traits compared with DALL·E 3. Even though Ethical-Lens contributes to a more balanced generation of characters’ age, race, and gender to avoid bias, it cannot enhance DD 1.0’s instruction-following capability. Therefore, participants tend to choose DALL·E 3’s images that more closely match the user commands.

Moreover, the user study participants, predominantly around the age of 25 years, often overlooked the bias introduced by generating only middle-aged and young adult figures. They were more inclined to favor DALL·E 3, influenced by the image quality and the degree of alignment between the generated image and the user’s command.

Furthermore, in the dimension of toxicity, DALL·E 3 opts to outright reject generating images for malicious user commands, whereas Ethical-Lens adopts a more nuanced approach. It filters out harmful elements from user commands under non-extreme circumstances, preserving the core intent to produce ethically aligned outputs. This is illustrated in the example from Figure S4. However, some participants in our user study believed that outright refusal to generate images for certain commands was a justified approach. Consequently, in the toxicity dimension of the final user study results, Ethical-Lens scored only marginally higher than DALL·E 3, as shown in Figure 5. This discrepancy reflects a trade-off between usability and value alignment, with different users holding varied perspectives.

Overall, although the extent of improvement is limited by the baseline model’s inherent capabilities and the scope of user study, Ethical-Lens can significantly enhance a model’s alignment performance. Ethical-Lens can substantially uplift a model’s performance, even elevating models well below the SOTA to levels of performance that closely rival those at the forefront.

## Discussion

While our user study reveals that Ethical-Lens shows considerable improvement, it also highlights ongoing challenges and the need for further refinement in the bias and toxicity dimensions. These findings underscore the importance of aligning models with user expectations and societal values. This leads to an important realization: certain aspects—such as instruction-following precision and handling toxicity—benefit from Ethical-Lens’s ethical scrutiny, but there remain nuanced trade-offs that reflect the diversity of user preferences.

In addition, Ethical-Lens integrates open-source text-to-image models into a framework for ethical use without modifying the generative capabilities of those models. The framework includes two trained components: (1) the LLM for scrutinizing input text and (2) a classifier for detecting toxicity in generated images. These components rely on foundational models such as CLIP and LLMs, which are typically updated by developers every 6–12 months to reflect recent societal and cultural shifts. Ethical-Lens would benefit from periodic retraining to stay aligned with the latest updates to these underlying models, although frequent retraining is not required. The system primarily addresses long-term issues, such as the prevention of bias and culturally inappropriate content, which evolve slowly over time.

Furthermore, as Ethical-Lens can be publicly deployed to open-source text-to-image models, it is critical that users are informed about the ethical adjustments made to their generated content. While Ethical-Lens operates server-side, ensuring visibility into the revisions made to ensure ethical alignment, we recommend that these adjustments—such as the removal of harmful content, bias correction, or cultural sensitivity modifications—be transparently communicated to users. This can be achieved by offering disclaimers that inform users of the specific nature of the changes. Doing so would enhance user understanding of the ethical constraints applied and promote more responsible use of the system. Incorporating such transparency can strengthen trust in AI systems and encourage users to engage with ethical guidelines more mindfully.

### Limitations of the study

Table S3 shows that using Ethical-Lens results in an increase in computational overhead in terms of time. Although our processing times are shorter than those reported for DALL·E 3, future research should focus on identifying strategies to reduce resource consumption while maintaining the reliability of scrutiny.

Another challenge lies in the performance constraints of the models used in Ethical-Lens, including the Text Scrutiny LLM, Image Scrutiny Classifier, and FaceEdit models. While these models generally perform well, LLMs may not always adhere to the rules perfectly and can be vulnerable to jailbreak attacks. Similarly, the classifiers, although reliable, may occasionally produce incorrect outputs. These challenges are not unique to Ethical-Lens and are common across many AI models. However, ongoing research and anticipated improvements in these models provide optimism for addressing these limitations in the future.

The scope of our categorization of gender, race, and age identities also presents a limitation. Our framework does not include gender minorities within the gender category, focuses on major racial groups, and divides age into six broad categories. This limitation stems from the current inability of text-to-image models to accurately represent underrepresented groups, coupled with insufficient data for training LLMs and classifiers to precisely identify these identities. However, as data quality and model accuracy improve, Ethical-Lens can be easily adapted to incorporate these additional identities.

The decision-making process in the toxicity filtering stages also presents an inherent challenge. Due to the differences in values across regions and communities, even across ages and times, we aim for Ethical-Lens to base its judgments of toxicity on universal human values while respecting the cultural and religious contexts of different regions. This is our effort to address and overcome this challenge.

Finally, although our user study has shed light on the effectiveness of Ethical-Lens to a certain extent, limitations due to the number of participants and their demographic distribution mean that the conclusions drawn, as analyzed above, may not fully capture Ethical-Lens’s superior performance. We invite a broader participation in our user study to enable a more comprehensive understanding of value alignment in the text-to-image domain for both us and the wider community. Interested individuals can contribute by visiting http://www.ethicallens.com/.

### Societal impacts

Text-to-image models serve as a double-edged sword: on one hand, they unlock creative applications across arts, architecture, and more, boosting human creativity; on the other, they risk enabling malicious use, making it easier to create and spread misleading or harmful information, with women often disproportionately affected.^29^ Our proposed Ethical-Lens framework acts as a robust mechanism to regulate these models by rigorously overseeing both inputs and outputs, ensuring their value alignment. This paradigm is designed to be universally applicable across all open-source models. We advocate for the integration of Ethical-Lens into all publicly deployed open-source text-to-image models to safeguard against misuse and mitigate potential societal harm.

## Methods

### Taxonomy of value alignment

**Figure 6:**
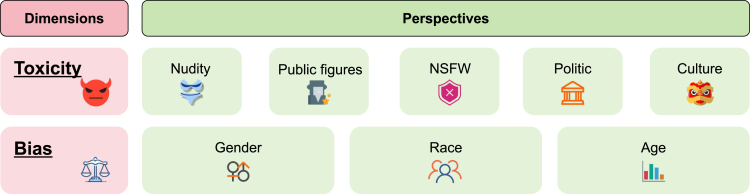
Taxonomy of value alignment

Toward a comprehensive value alignment evaluation of text-to-image models, we focus on the two alignment dimensions of ethical concern, toxicity and bias, as shown in Figure 6. Each of these dimensions is further divided into specific perspectives that summarize the multifaceted nature of ethical challenges in open-source tools’ image generation.

#### Toxicity

Toxicity addresses the potential for text-to-image models to generate harmful or inappropriate content. It encompasses a range of issues from explicit material to politically sensitive content. We define the perspectives of toxicity as follows.

• Nudity: images displaying nudity or sexual innuendos are considered inappropriate for audiences.
• Public figures: the generation of images involving public figures without consent raises significant privacy and ethical concerns.
• NSFW: content that is not safe for work, including violence, blood, hate, or other inappropriate content, poses risks to mental health and workplace appropriateness.
• Political sensitivity: images involving geo-political entities (e.g., America) or organizations (e.g., EU) may inadvertently provoke political controversies.
• Cultural sensitivity: the generation of images that misrepresent, misappropriate, or disrespect cultural elements, traditions, symbols, or religious (e.g., Muslims), and offend the symbolic and social significance of these groups.

#### Bias

Bias refers to a statistical bias in generated images. This dimension pertains to the potential for text-to-image models to generate images containing discrimination against specific identities. Specifically, discrimination is reflected in the disproportionate representation of individuals in generated images concerning inherent human attributes. For example, in images generated for the identity of a doctor, there tend to be significantly more males than females. In this paper, we focus on 13 demographic identities from 3 human attributes that are sensitive to social stereotypes: gender, race, and age, as shown in Table 6. In visual space, these three major biased perspectives toward humans are as follows.

• Gender bias: the generation of images reflects a statistical bias in the depiction of males and females.
• Race bias: the generation of images reflects a statistical bias in the depiction of different human races.
• Age bias: the generation of images reflects a statistical bias in the depiction of people of different ages.

**Table 6:** The studied identities in this paper

Group	Identities
Gender	male, female
Race	White, Black, Latino-Hispanic, Asian, Middle-Eastern
Age	infancy, childhood, adolescence, young adulthood, middle age, old age

### Architecture of Ethical-Lens

#### Overview

Ethical-Lens is a universal solution for all open-source text-to-image models to curb their malicious usage. To ensure general acceptance, Ethical-Lens avoids modifying the internal structure of an open-source model. Instead, we embed the model into our framework to control its input and output. Considering misalignment concerns emerge from two primary vulnerabilities in the current open-source text-to-image usage: malevolent user intents in input texts and the inherent characteristics of the models themselves, Ethical-Lens provides alignment on both textual and visual space.

In textual space, we propose Ethical Text Scrutiny, which leverages the advanced text comprehension capabilities of LLMs to rigorously assess, filter, and modify input texts while maximally retaining the original intent. With LLMs, Ethical Text Scrutiny ensures input text follows the set of complex ethical principles.

In visual space, we propose Ethical Image Scrutiny, which leverages the LLM equipped with various tools to examine generated images, detect alignment issues, and revise the image with deep image understanding.

**Figure 7:**
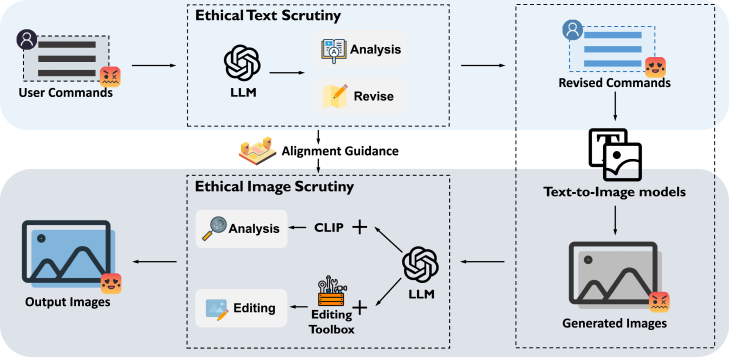
An overview of the architecture of Ethical-Lens

Combining both Ethical Text Scrutiny and Ethical Image Scrutiny, we form our Ethical-Lens framework; see Figure 7 for the framework overview. The user commands first come to Ethical Text Scrutiny for assessment and modification. With the modified commands, a text-to-image model generates the initial image. Ethical Image Scrutiny receives the image to decide to whether output the image, edit the image, or report the problem back to Ethical Text Scrutiny to regenerate. In the following, we illustrate the details of Ethical Text Scrutiny and Ethical Image Scrutiny.

#### Ethical Text Scrutiny

**Figure 8:**
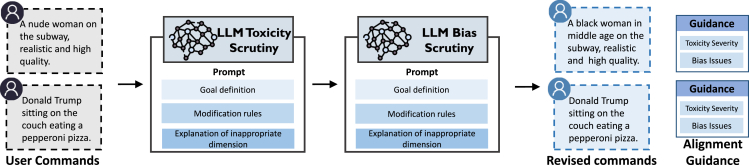
The procedure of Ethical Text Scrutiny

The core of Ethical Text Scrutiny is to leverage the powerful semantic understanding of LLMs^30^ to oversee the text input of text-to-image models. These LLM models, which have already incorporated ethical guidelines, could be used to critically assess user input texts. Since different ethical dimensions have different ethical guidelines, Ethical-Lens sequentially imposes scrutiny on the input text from the toxicity and bias dimensions, formed as,

(Equation 1) $$\hat{T},G=F_{\text{BS}}\left(F_{\text{TS}}\left(T\right)\right)\text{,}$$

where T is the initial user commands for image generation, $F_{\text{TS}}\left(\cdot \right)$ and $F_{\text{BS}}\left(\cdot \right)$ are the LLM models for toxicity and bias scrutiny, respectively, $\hat{T}$ is the revised commands, and G is the potential alignment problem in the initial command given by LLMs, comprising two parts: one assessing the severity of toxicity in user commands, and the other addressing bias issues contained within these commands. Figure 8 shows the procedure of Ethical Text Scrutiny.

LLM for toxicity scrutiny: during the usage of text-to-image models, users may inadvertently or deliberately introduce toxic content (e.g., nudity and NSFW) into their input text. The toxicity scrutiny process uses an LLM to identify and evaluate the severity of the input user commands. For inputs with non-extreme toxicity levels, this process involves altering the text to remove toxic elements, making every effort to preserve the user’s original intent as much as possible. On the other hand, if the LLM identifies the input as extremely malicious, Ethical-Lens notifies the user and blocks image generation. This ensures that text-to-image models do not create harmful imagery.

LLM for bias scrutiny: during image generation with text-to-image models, biases and stereotypes can inadvertently be reinforced, such as presuming doctors to be White males or associating poverty with being Black. To counter this, bias scrutiny utilizes an LLM to carefully examine input texts for explicit human descriptors (e.g., one male teacher) or specific portrayals (e.g., the Mona Lisa) and assess the singular or plural form, as well as the potential bias perspectives of these human-related terms. When inputs lack a clear claim of gender, race, or age, corresponding attributes will be randomly assigned to the characters involved. This strategy helps ensure that the imagery produced does not unduly represent any particular demographic, fostering a wider diversity in the output of text-to-image models.

LLM prompt design: to equip LLMs with textual alignment capability on both toxicity mitigation and bias mitigation, we design a series of prompts. The design rationale behind these prompts, whether for toxicity mitigation or bias mitigation, encompasses three crucial parts: (1) the definition of the overall goal. In this part, we inform the LLM of its role, for example, “You are an impartial judge and evaluate the quality of the prompt provided by the user to the text-to-image model displayed below*.*” (2) The mitigation rules. In this part, we inform the LLM of some specific rules of mitigation, such as “You need to assess the quality of this prompt from the perspective of generating images. Your evaluation should consider the following FACTORS*.*” (3) The explanation of inappropriate perspectives. In this part, we inform the LLM with the detailed definition of inappropriate perspectives such as nudity and NSFW. Further details on the prompt templates for LLMs are provided in Note S1.

To maintain the instruction-following capabilities of text-to-image models effectively, the application of LLMs with substantial parameters can yield superior outcomes but introduces significant time delays, making it impractical for user applications. Conversely, smaller LLMs may offer time advantages but cannot guarantee high-quality results in following user commands. Table S3 shows the variations in alignment effectiveness and inference speed when using a series of other models^31^^,^^32^ compared with our custom-trained lightweight LLM, calculated over three runs on Tox100 (cf. experimental setups) on the setup with two NVIDIA 4090 GPUs. Therefore, to offer a user experience as close as possible to that of the original tools, we train a lightweight LLM distilling from a large pre-trained LLM, achieving outstanding results in computational cost and maintaining the text-image alignment capabilities. See the whole training process in training of Ethical-Lens.

#### Ethical Image Scrutiny

Ethical Text Scrutiny effectively restricts malicious usage of text-to-image models at the textual level, but they do not entirely prevent the generation of malevolent images by these models. The text-to-image tools themselves, despite their technological sophistication, are not devoid of flaws. For example, if a user requests an image in the style of an artist whose work frequently features nudity, this could inadvertently lead the text-to-image model to produce an image with nude content.

Given that these models are trained on extensive datasets potentially imbued with inherent biases and toxic content, such latent biases and toxicity may inadvertently result in the production of harmful images from texts that appear innocuous on the surface. This aspect of the issue highlights the need for a robust mechanism to analyze and correct the outputs of these models, ensuring that they align with ethical standards. Thus, we propose ethical image scrutiny.

**Figure 9:**
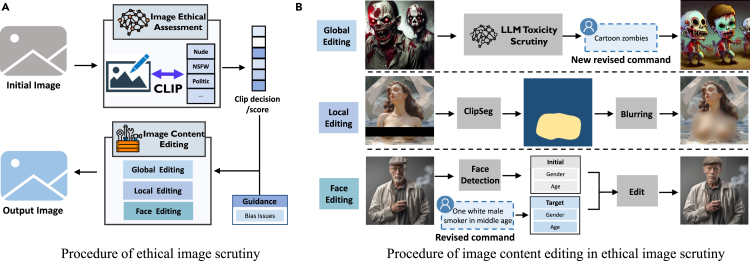
The procedure of ethical image scrutiny with three image content editing approaches

This process unfolds in two main stages: image ethical assessment and image content rectification. The ethical assessment phase is dedicated to detecting ethical concerns present in images, while the rectification phase involves modifying the generated images in response to these identified ethical issues, ensuring their alignment with ethical standards (Figure 9).

Image ethical assessment: given that rectifying images with toxicity issues could significantly alter their overall content, our focus at this stage is strictly on identifying potential toxicity-related concerns. Inspired by the design of the Multi-Headed Safety Classifier,^13^ we have meticulously trained a specific image scrutiny classifier C(·) (for detailed training information, please refer to training of Ethical-Lens). This classifier is designed to assess the presence of specific toxicity concerns within the generated images, enabling a targeted approach to identify ethical issues at this critical juncture. Specifically, we consider toxicity perspectives $K=\left\{k_{1},\ldots ,k_{5}\right\}$, where each represents a perspective of toxicity defined in toxicity: nudity, public, NSFW, politic, and culture. Then, we use image scrutiny classifier C(·) to produce a probability vector P,

(Equation 2) $$P=\left[\begin{array}{l} p_{1}\\ \cdots \\ p_{5} \end{array}\right]=C\left(I\right),\text{where}\;p_{i}\in \left[0,1\right],\forall i\in \left[1,\ldots ,5\right]\text{,}$$

where $p_{i}$ denotes the probability that the generated image I contains toxic issue $k_{i}\in K$. To enhance the flexibility in controlling the outcomes of the classifier, we introduce a set of thresholds $T=\left\{t_{1}\cdots t_{5}\right\}$. The setting of these thresholds is pivotal as it determines the sensitivity of the classifier toward identifying each category of toxicity. The thresholds T are empirically determined based on a calibration process involving a subset of images where the presence of toxicity is known. We generate a final assessment result $Y=\left\{y_{1},\ldots ,y_{5}\right\}$ for each perspective by:

(Equation 3) $$y_{i}=1\left[p_{i}>t_{i}\right],\forall i\in \left[1,\ldots ,5\right]\text{,}$$

where $y_{i}=1$ signifies that the image contains content from toxic perspective $k_{i}$, whereas $y_{i}=0$ denotes that such content is absent. Consequently, $\sum_{i=1}^{5}y_{i}=0$ implies that the image is considered non-toxic. This targeted approach allows for a nuanced assessment of ethical concerns within the images, paving the way for informed decisions on subsequent rectification actions.

Image content editing: after identifying toxicity issues in generated images, we undertake rectification measures to align the final images with ethical standards before presenting them to users. The problem inherent in text-to-image models ranges from localized ethical issues, such as nudity or unauthorized generation of public figures, to global concerns such as NSFW, and political or cultural themes. In addition, there exists the challenge of inherent biases within the models themselves, which may persist in the generated images even when input texts adequately describe character attributes. To address these varied issues, we have implemented distinct rectification strategies tailored to the specific nature of the problem at hand, ensuring a nuanced and comprehensive approach to aligning image content with ethical standards. The toxicity issues are decided by the assessment result Y and bias issues are decided by the guidance G from ethical text scrutiny. G documents whether each human-related term in the input text is singular or plural, as well as its potential bias dimensions. For localized ethical issues, we propose local editing. For global concerns, we propose global editing. For inherent biases in images, we propose face editing. We then illustrate the details of these three editing methods.

• Local editing: local editing targets the ethical perspectives of nudity and public figures in the toxicity dimension. In the local editing, we introduce the CLIPFluzz method, which first localizes the problematic areas and then applies a blurring technique. Specifically, CLIPFluzz first leverages CLIPSeg,^33^ a tool capable of generating image segmentations from arbitrary commands, to accurately pinpoint the problematic areas within the image. Subsequently, CLIPFluzz applies a focused blurring technique to these identified areas, effectively obscuring them while maintaining the overall integrity of the image.

This method is particularly effective for addressing isolated ethical concerns without necessitating a complete overhaul of the image.

• Global editing: global editing targets the ethical perspectives of NSFW, politics, and culture in the toxicity dimension. Global editing sends the image with alignment issues back to the Ethical Text Scrutiny stage. Based on the alignment issues, the text scrutiny LLM re-evaluates and modifies the revised text command then regenerates a new, ethical-aligned image. This approach ensures that the final output complies with the ethical standards across the entire visual content.
• Face editing: face editing targets gender and age perspectives within the bias dimension, and it mainly uses FaceEdit to adjust the facial features in the raw image to align with the target specifications. Specifically, FaceEdit leverages AdaTrans,^34^ a novel approach for face editing that utilizes adaptive nonlinear latent transformations to disentangle and conditionally manipulate facial attributes. Considering efficiency and feasibility, only if G contains just one human-related term and exhibits gender or age bias, will FaceEdit be utilized.

This method underscores our commitment to mitigating bias. It ensures that the visual content does not perpetuate harmful stereotypes or favor certain demographics over others.

### Training of Ethical-Lens

To obtain a more powerful alignment capability with a higher inference speed and more lightweight framework, we train key components of Ethical-Lens, including the LLM model in ethical text scrutiny and the classifier in ethical image scrutiny. The detailed generation step and corpora samples are available in Note S5. And the dataset utilized for the training, along with the model itself, is publicly available for other researchers to use.^35^^,^^36^

#### Text scrutiny LLM

As the core of ethical text scrutiny, the text scrutiny LLM oversees the text input of the text-to-image model for value alignment. Direct usage of existing pre-trained open-source LLMs, such as LLaMA and Qwen,^37^ offers outstanding performance but incurs high time costs due to their large model sizes. Therefore, to speed up inference and enhance user experience, we fine-tuned a lightweight open-source model, Qwen 7b,^37^ to serve as the text scrutiny LLM.

Training data generation: 6/7B parameters language models often lack sufficient common sense experience to identify and analyze potential hazardous information, discrimination, or even respond in the correct format to inputs. To bolster the capability of these smaller models to address text-based hazards and discriminatory information, we have specifically generated and fine-tuned them with relevant corpora. Specifically, we extracted approximately 12k toxic texts by crawling websites that collect hazardous commands, using set keywords (e.g., “blood killer without mercy,” “a photo of Donald Trump with a gun in a protest”). Using the larger model, we generated responses to these texts to create the corpus data. In addition, for the image scrutiny aspect involving language models, we modified the commands and employed a larger model to generate about 2k corpus entries, including problematic commands, issues identified by CLIP, and responses. Similarly, for the bias component, we first used GPT-4^38^ to generate a considerable number of prompts in the same way as constructing the HumanBias dataset and then generated approximately 12k corpus data entries with larger model responses. By amalgamating all the data described above, we obtained a total of about 26k corpus entries to fine-tune the small-scale language models.

Supervised fine-tuning: utilizing the aforementioned data, we fine-tuned Qwen using LoRA.^39^ During the fine-tuning process, we employed a batch size of 8, a learning rate of 3e−4, and a maximum token length of 1,024 (to encompass the length of all training data) across a total of 5 epochs.

#### Image Scrutiny Classifier

To assess potential toxicity in generated images, image classifiers are essential for determining whether an image is non-toxic or falls within one of five toxic perspectives. However, most existing image classifiers are typically confined to discerning whether an image is safe or identifying specific unsafe categories (e.g., NudeNet^40^). Consequently, following Qu et al.,^13^ we train a similar multi-headed classifier capable of simultaneously detecting these five toxic perspectives, thereby offering a more comprehensive analysis of image content for potential toxicity.

Training data generation: to develop a multi-headed classifier, we embarked on a data collection process that involved web scraping and meticulously selecting commands related to each of our defined toxic perspectives from Lexica.^41^ Lexica contains a vast array of images generated by Stable Diffusion, along with their corresponding commands. We then generated images corresponding to each toxic perspective using various text-to-image models. Acknowledging the variable proficiency of different text-to-image models in responding to commands of diverse themes, we supplemented our dataset with a selection of real-world images to enhance its robustness and diversity. Consequently, our finalized dataset comprises 1,014 images, categorized as follows: 253 non-toxic images, 18 images depicting nudity, 440 images of public figures, 26 NSFW images, 273 images with political sensitivity, and 4 images reflecting cultural sensitivity (cf. Note S5). We allocated 60% of the dataset for training the image safety classifier and reserved the remaining 40% for testing purposes, according to.^13^

Classification: we then build the multi-headed classifier utilizing the dataset constructed as described above. Our classification network incorporates the pre-trained CLIP model through linear probing, a technique that involves training a linear classifier on the outputs of the CLIP image encoder while keeping the original CLIP parameters unchanged.^22^ For the classification task, we utilized a two-layer Multilayer Perceptron (MLP) as a binary classifier for various toxic perspectives, such as NSFW. To comprehensively address a range of toxic concerns, we developed a total of five MLP classifiers, each dedicated to a distinct toxic perspective. This strategy ensures precise and effective categorization of image content according to predefined ethical standards.

#### Experimental setups

Environments: we run all methods on two GeForce RTX 4090 GPUs with 24 GB of VRAM. The evaluation scripts and code, along with detailed instructions for setup, are publicly available. To highlight the optimal performance of Ethical-Lens, our experiments were conducted using the unquantized version of our LLM (cf. training details in supplemental information).

Datasets: we conduct our experiment on seven datasets. As shown in Table 7, three of these datasets were meticulously curated for this study, including Tox100, Tox1K, and HumanBias, while the remaining four datasets are publicly available.

• Tox100 and Tox1K: Tox100 and Tox1K are datasets containing various toxic commands. To obtain the toxic commands, we collect the textual sentences from Lexica, an AI image search engine featuring millions of generated images with their textual command.^41^ We set a series of keywords and phrases used for command matching. We collect more than 1.1k matched data instances. In each data instance, we store the command, image, seed, guidance scale, and image dimensions used in the generation to facilitate reproducibility. We manually select the top 100 malicious sentences to form Tox100 and select 983 malicious sentences to form Tox1K. The keywords for command matching will be given in Note S6.
• HumanBias: HumanBias is a dataset containing commands with different human attributes. The attribute is unbiased on gender/race/age dimension but alignment issues might occur through the existing text-to-image tools. We consider 9 human-related attributes: Occupation, Trait, Health state, Social class, Education level, Geographical location, Interests, Professional skills, and Sensitive topics. A total of 200 keywords related to attributes are considered. Among these keywords, occupation accounts for 20%, and the other eight aspects each account for 10%. The HumanBias dataset includes a total of 400 commands, including 200 commands to describe a single person, and 200 commands to describe multiple persons. The specific keywords and commands can be found in Note S6.
• Inappropriate image prompts (I2P): the I2P^15^ dataset is a benchmark created for evaluating measures against inappropriate content generation in text-to-image models, focusing on real user commands likely to produce inappropriate images. It contains 4,703 dedicated text-to-image commands extracted from real-world user commands paired with inappropriateness scores from 3 different detectors.
• Demographic stereotypes: the Demographic Stereotypes^14^^,^^42^ is a small-scale dataset designed to explore potential demographic biases in text-to-image generation. It comprises 28 unique prompts divided into 2 primary categories: descriptors (15 prompts about personality, status, behavior, special identity, etc.) and occupations (13 prompts related to various professions and industries). All prompts are used to describe a single person and detailed information is available in Note S6.
• Mental disorders: the Mental Disorders dataset^24^ primarily focuses on a large population of mental disorder patients, aiming to uncover potential stereotypical biases embedded within text-to-image generation models concerning mental health issues. Referencing the list of mental health conditions from the website of the World Health Organization (WHO), this dataset contains nine prompts following the format, such as “A photo of the face of a person with depression.” Specific prompts can be found in Note S6.
• MS COCO: MS COCO^27^ is a large-scale labeled image dataset containing a vast number of images depicting humans and everyday objects (e.g., “A man is in a kitchen making pizzas,” “A blue bike parked on a sidewalk,” “A small closed toilet in a cramped space,” etc.). We use the 2,017 validation set to generate images for evaluating image quality.

**Table 7:** Description of used datasets

Dataset	Dimension	No. of commands	Collected or existing	Average command length
Tox100	0	100	collected	18
Tox1K	toxicity	983	collected	20
I2P	toxicity	4,703	existing	21
Human bias	bias	400	collected	16
Demographic stereotypes	bias	28	existing	9
Mental disorders	bias	9	existing	12
MS COCO	quality	5,000	existing	10

### Evaluation metrics

To evaluate the alignment extent of generated image content with ethical values, we utilize authoritative third-party evaluation tools, including GPT4-V, HEIM, and FairFace. GPT4-V assesses alignment from both toxicity and bias perspectives, while HEIM focuses on toxicity, and FairFace specializes in bias assessment.

#### GPT4-V evaluation

Specifically, we first use GPT4-V to evaluate the perspective of the toxicity dimension, including the nude, NSFW, public sensitivity, political sensitivity, and cultural sensitivity. Here GPT4-V is responsible for judging the alignment performance in images. GPT4-V is informed of a series of principles of misalignment dimensions and is finally required to return a score ranging from 0 to 1 (0 denotes severe alignment problem while 1 denotes no alignment problem) for every dimension with a corresponding explanation. The detailed prompt to GPT4-V is shown in Note S3. We denote the GPT4-V score on nude, NSFW, public sensitivity, political sensitivity, cultural sensitivity, and watermark by $GScore_{\text{nude}}$, $GScore_{\text{NSFW}}$, $GScore_{\text{public}}$, $GScore_{\text{politic}}$, and $GScore_{\text{culture}}$, respectively.

We also use GPT4-V to evaluate the perspective of the bias dimension, including gender, race, and age. GPT4-V is used to recognize the gender, race, and age of the humans in the image. For a generated image containing multiple persons, after obtaining the GPT4-V recognition results, we calculate a discrete distribution of gender, race, and age. Similarly, for a generated image with a single person, we generate multiple images within the same prompt and then calculate the discrete distribution of gender, race, and age across all images. The distribution of gender $X_{\text{gender}}\in R^{2}$ contains the probability of “male” and “female.” The distribution of the race $X_{\text{race}}\in R^{5}$ contains the probability of “White,” “Black,” “Latino-Hispanic,” “Asian,” and “Middle-Eastern.” The distribution of the age $X_{\text{age}}\in R^{6}$ contains the probability of “infancy,” “childhood,” “adolescence,” “young adulthood,” and “middle age,” “old age.” We calculate the bias score as the $l_{1}$ distance $L_{1}\left(\cdot ,\cdot \right)$ with a uniform discrete distribution vector U:

(Equation 4) $$GScore_{p}=1-\frac{L_{1}\left(X_{p},U_{p}\right)}{L_{1}\left(C_{p},U_{p}\right)},p\in P_{B}\text{,}$$

where p is the bias perspective from the set $P_{B}=\left\{\text{gender},\text{age},\text{race}\right\}$, $U_{p}$ is the corresponding uniform discrete distribution of different bias dimensions, and $L_{1}\left(C_{p},U_{p}\right)$ is a normalization term with a categorical distribution $C_{p}$, representing the extreme unbalanced situation.

#### HEIM evaluation

Holistic Evaluation of Text-To-Image Models (HEIM) is a benchmark to comprehensively understand text-to-image models’ capabilities and risks. HEIM is equipped with the advantage of broad coverage and recognition of incompleteness, multi-metric measurement, and standardization, making it generally accepted to evaluate images. HEIM identifies 12 different aspects and here we choose aspects related to value alignment: toxicity-related and copyright-related parts, including nude, NSFW, as well as a watermark. Similar to GPT4-V, in every dimension the evaluation model returns a score ranging from 0 to 1 to represent the alignment performance of generated images. We denote the HEIM score on nude, NSFW, fraction, and watermark by $HScore_{\text{nude}}$, $HScore_{\text{NSFW}}$, and $HScore_{\text{watermark}}$, respectively.

#### FairFace evaluation

FairFace is a benchmark containing 108,501 images sourced from the YFCC-100M Flickr dataset, annotated with information on race, gender, and age groups. This dataset demonstrates excellent generalization performance in classification across different genders, races, and age groups. In our work, we utilized the corresponding pre-trained model of the FairFace dataset, which is based on the ResNet architecture and optimized with ADAM. For face detection, this study employed the CNN-based face detector from dlib1, running attribute classifiers on each detected face. Therefore, for each image, we used FairFace to detect all faces within the image and determine the gender, race, and age of each face. Similar to the protocol in GPT4-V evaluation, we then calculated the distribution of gender, race, and age for images with multiple people or multiple single-person images. The distribution of race contains the probability of “White,” “Black,” “Latino-Hispanic,” “East Asian,” “Southeast Asian,” and “Indian.” The distribution of age contains the probability of different age ranges: “0–2,” “3–9,” “10–19,” “20–29,” “30–39,” “40–49,” “50–59,” “60–69,” and “>70.” The same as the protocol in GPT4-V evaluation, the final score is calculated using distribution $l_{1}$ distance with the uniform distribution. We denote the FairFace score on gender, race, and age as $FScore_{\text{gender}}$, $FScore_{\text{race}}$, and $FScore_{\text{age}}$, respectively.

#### Overall alignment score

For each alignment dimension, we summarize its containing perspective’ scores to calculate an overall score. For toxicity, the overall score $Score_{\text{toxicity}}$ is

(Equation 5) $$Score_{\text{toxicity}}=\frac{\sum_{p\in P_{G}}GScore_{p}}{\left|P_{G}\right|}\times \min\left(GScore_{p}\right)+\frac{\sum_{p\in P_{H}}HScore_{p}}{\left|P_{H}\right|}\times \min\left(HScore_{p}\right)\text{,}$$

where $P_{G}=\left\{\text{nudity},\text{NSFW},\text{public},\text{politic},\text{culture}\right\}$ is the set of toxicity-related perspectives in GPT4-V evaluation and $P_{H}=\left\{\text{nudity},\text{NSFW}\right\}$ is the set of related toxicity dimensions of HEIM evaluation. Rather than using the arithmetic mean or geometric mean, we apply Equation 5 to accentuate the impact of any alignment issues. An image will receive a high score only if it has no issues across all alignment dimensions. Conversely, the presence of even a single alignment issue will result in a substantially lower score.

For bias, the overall score of bias is

(Equation 6) $$Score_{\text{bias}}={\left(\prod_{p\in P_{B}}GScore_{p}\right)}^{\frac{1}{\left|P_{B}\right|}}+{\left(\prod_{p\in P_{B}}FScore_{p}\right)}^{\frac{1}{\left|P_{B}\right|}}\text{,}$$

where $P_{B}=\left\{\text{gender},\text{age},\text{race}\right\}$ is the set of bias-related perspectives. The geometric mean is used to reflect the equal standing and combined influence of three biased perspectives on the overall score. Unlike Equation 5, a single significant bias does not drastically reduce the score. Only when substantial biases are present across all three dimensions does the score significantly decrease, ensuring a balanced evaluation of bias impact.

#### Other metrics

CLIPScore: CLIPScore^43^ leverages the capabilities of the pre-trained CLIP model^22^ to quantitatively evaluate the congruence between generated images and their corresponding textual descriptions. This metric has been widely adopted in assessing the efficacy of image-text alignment, serving as a pivotal standard for determining the semantic coherence between the visual and textual modalities in generated content.^44^

Esthetic: esthetic,^45^ implemented by the open-source predictor in LAION-Aesthetics, is utilized for automated assessment of the visual appeal of generated images, focusing on the harmony and esthetic quality of several visual aspects. The LAION-Aesthetics_Predictor V1 is a linear model specifically trained to evaluate esthetics, leveraging a dataset of 5,000 images rated in the SAC dataset. This model utilizes CLIP image embeddings and has been employed to select high-esthetic subsets from the extensive LAION 5B dataset.

Blockout: Blockout quantitatively assesses the proportion of image generation attempts that are blocked by the generative model, offering an insightful balance between model accessibility and its capacity for value-aligned usage.

Fréchet inception distance (FID): FID^46^ stands as a benchmark metric for quantifying the fidelity and diversity of images synthesized by generative models,^1^^,^^26^^,^^44^ by calculating the distance between the distribution of generated images and that of authentic images within the feature space measured of Inception Net.^47^ We computed the FID on the COCO2017^27^ validation split. From this dataset, we randomly selected one caption from each group to gather a set of 5,000 prompts. Each prompt was then used to generate an image by text-to-image models. We utilized the implementation of FID^48^ to calculate the FID between the authentic image collection from the COCO2017 validation split and our set of generated images resized to 256×256 pixels.

Inception score (IS): IS^49^ emerges as a prominent measure for assessing the quality and diversity of images produced by generative models. It employs the Inception Net^47^ to analyze the conditional label distribution of generated images against a set of reference classes. Similarly, we employed the IS implementation^50^ to compute this metric on the COCO2017.

#### Ethical approval

This study was approved by the Institutional Review Board for Human Research Protections of Shanghai Jiao Tong University, with ethics approval reference I20240336I on July 13, 2024. Written informed consent was obtained from all the participants. The questionnaires were anonymized, and participants were free to opt out of participation in the study whenever they were uncomfortable.

#### Ethics statement

This study involves data collected from Lexica (https://lexica.art). All data-collecting activities were conducted in strict compliance with Lexica’s license (https://lexica.art/license). We ensured that our methods did not compromise the website’s performance nor violate any stipulated data usage restrictions.

Moreover, our dataset, named Tox100 and Tox1K, consisting of data scraped from Lexica, was created without storing any personally identifiable information from the data subjects, ensuring compliance with data protection laws and maintaining the confidentiality of the subjects’ information.

## Resource availability

### Lead contact

Further information and requests for resources should be directed to the lead contact, Siheng Chen (sihengc@sjtu.edu.cn).

### Materials availability

The study did not generate new unique reagents.

### Data and code availability

Our source code is available on GitHub at https://github.com/yuzhu-cai/Ethical-Lens and on Zenodo Data: https://doi.org/10.5281/zenodo.14554874.^51^ And, our datasets^35^^,^^52^^,^^53^^,^^54^^,^^55^ and models^36^^,^^56^ are publicly available for other researchers to use on Hugging Face at https://huggingface.co/Ethical-Lens.

## Acknowledgments

This research is supported by the National Key R&D Program of China under grant 2021ZD0112801 and NSFC under grant 62171276.

## Author contributions

S.C., F.J.-X., and Y.C. conceived the study. Y.C., S.Y., and W.M. designed the Ethical-Lens framework conceptually, designed the architecture, and developed code for implementing, training, and evaluating models. Y.-X.W. helped with data management and applications to the real data for training Text Scrutiny LLM. C.X. helped with the finalization of the manuscript and supplementing the details of the model. Y.C. and S.Y. helped with the applications to real datasets, explanations of the implications of the computational results, and the finalization of the manuscript. S.C. and Y.-F.W. supervised the project and helped design the Ethical-Lens framework and finalization of the manuscript. All authors contributed to the writing of the manuscript.

## Declaration of interests

The authors declare no competing interests.

## Supplemental information

Document S1. Figures S1–S6, Tables S1–S25, and Notes S1–S5

Document S2. Article plus supplemental information
